# Regulation of immune responses to infection through interaction between stem cell-derived exosomes and toll-like receptors mediated by microRNA cargoes

**DOI:** 10.3389/fcimb.2024.1384420

**Published:** 2024-05-02

**Authors:** Mehrdad Moosazadeh Moghaddam, Elham Behzadi, Hamid Sedighian, Zoleikha Goleij, Reza Kachuei, Mohammad Heiat, Abbas Ali Imani Fooladi

**Affiliations:** ^1^ Tissue Engineering and Regenerative Medicine Research Center, Baqiyatallah University of Medical Sciences, Tehran, Iran; ^2^ The Academy of Medical Sciences of I.R. Iran, Tehran, Iran; ^3^ Applied Microbiology Research Center, Systems Biology and Poisonings Institute, Baqiyatallah University of Medical Sciences, Tehran, Iran; ^4^ Molecular Biology Research Center, Systems Biology and Poisonings Institute, Baqiyatallah University of Medical Sciences, Tehran, Iran; ^5^ Baqiyatallah Research Center for Gastroenterology and Liver Diseases (BRCGL), Baqiyatallah University of Medical Sciences, Tehran, Iran

**Keywords:** microbial infection, stem cell-derived exosomes, toll-like receptors, immunomodulation, host-directed therapy

## Abstract

Infectious diseases are among the factors that account for a significant proportion of disease-related deaths worldwide. The primary treatment approach to combat microbial infections is the use of antibiotics. However, the widespread use of these drugs over the past two decades has led to the emergence of resistant microbial species, making the control of microbial infections a serious challenge. One of the most important solutions in the field of combating infectious diseases is the regulation of the host’s defense system. Toll-like receptors (TLRs) play a crucial role in the first primary defense against pathogens by identifying harmful endogenous molecules released from dying cells and damaged tissues as well as invading microbial agents. Therefore, they play an important role in communicating and regulating innate and adaptive immunity. Of course, excessive activation of TLRs can lead to disruption of immune homeostasis and increase the risk of inflammatory reactions. Targeting TLR signaling pathways has emerged as a new therapeutic approach for infectious diseases based on host-directed therapy (HDT). In recent years, stem cell-derived exosomes have received significant attention as factors regulating the immune system. The regulation effects of exosomes on the immune system are based on the HDT strategy, which is due to their cargoes. In general, the mechanism of action of stem cell-derived exosomes in HDT is by regulating and modulating immunity, promoting tissue regeneration, and reducing host toxicity. One of their most important cargoes is microRNAs, which have been shown to play a significant role in regulating immunity through TLRs. This review investigates the therapeutic properties of stem cell-derived exosomes in combating infections through the interaction between exosomal microRNAs and Toll-like receptors.

## Introduction

**Figure 1:**
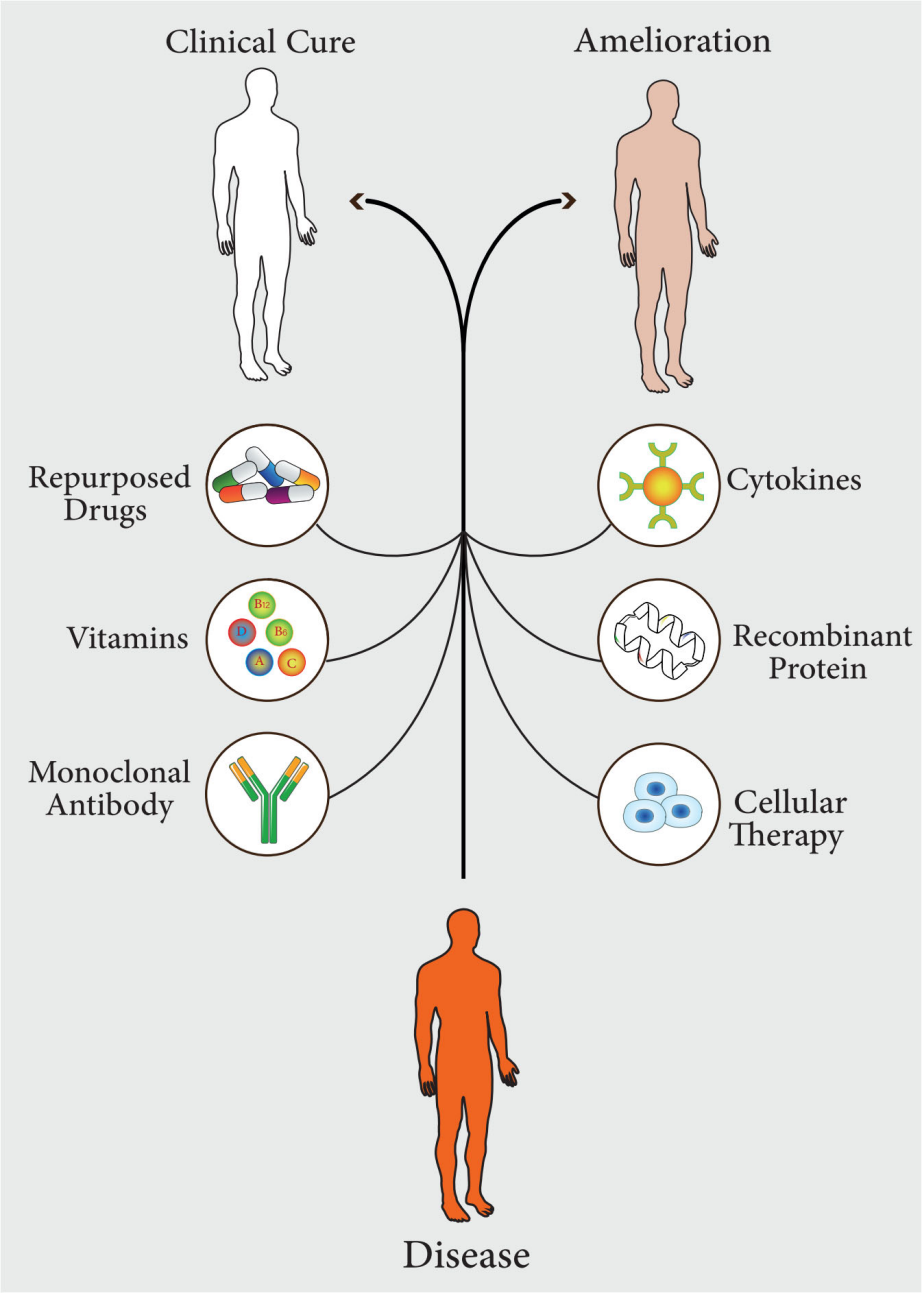
Different pharmaceutical agents, which can be used in host-directed therapy (HDT). These approaches lead to complete treatment of the disease or improvement of symptoms.

In recent years, the widespread and uncontrolled use of antibiotics has led to the spread of antibiotic-resistant strains. This resistance to conventional antibiotics has led to a significant increase in mortality from infectious diseases (Moravej et al., 2018; Tang KWK. et al., 2023). Some scientific reports show that infectious diseases will be the leading cause of death by 2050 (de Kraker et al., 2016). Therefore, there is a need for newer approaches to improve treatment procedures to minimize the significant morbidity and mortality associated with infectious diseases (Moghaddam et al., 2015). Recent studies on pathogen-host interactions, pathogenesis, host innate and adaptive immune responses, and inflammatory pathways have led to the identification and development of diverse and comprehensive approaches based on host-directed therapy, which have now become useful strategies for the treatment of infectious diseases (Varma et al., 2020). In general, host-directed therapy involves the use of products that alone or simultaneously can result in strengthening host defense mechanisms or reducing excessive inflammation, leading to improved clinical outcomes by reducing complications, mortality, and organ injury, and improving their long-term function (Kaufmann et al., 2018). This treatment approach includes pharmaceutical agents with good safety profiles, immunomodulatory agents, biological components such as monoclonal antibodies, cytokines and vitamins, nutritional supplements, and cell therapy or cell products (**Figure 1**) (Zumla et al., 2016). Host-directed therapies can increase the efficiency of immune responses in three ways, which include improving the host’s cellular responses to pathogens, targeting virulence factors that cause disease, and activating immunological memory and innate and adaptive immune responses (Zumla et al., 2016; Wallis et al., 2023).

The innate immune system serves as a rapid defense mechanism against invading microbes and enables the adaptive immune system to respond to pathogen-specific antigens (Marshall et al., 2018). Activation of the innate immune system for a short time is beneficial as it triggers cellular protective mechanisms for tissue repair. However, prolonged or excessive activity of immune system is harmful and can lead to irreversible changes in organ structure and function (Warrington et al., 2011; Riera Romo et al., 2016). Toll-like receptors (TLRs) are a family of pattern recognition receptors (PRRs) that play a crucial role in detecting microbial invasions and triggering innate and adaptive immune responses, making them a key component of the innate immune responses (Fitzgerald and Kagan, 2020; Li and Wu, 2021). PRRs are thought to function as germline-encoded proteins that identify conserved microbial products called pathogen-associated molecular patterns (PAMPs), thereby inducing activities that stimulate host immunity and defense (Suresh and Mosser, 2013). In general, activation of PRRs can be caused by PAMPs and damage-associated molecular patterns (DAMPs) trigger cellular signaling cascades through mediators such as TLRs (Amarante-Mendes et al., 2018; Zindel and Kubes, 2020). PAMP compounds are not present in the host and are also called microbe-associated molecular patterns (MAMPS). DAMPs known as alarmins, are endogenous components that are often released by stressed or injured cells. They can be classified as protein damage-associated molecular patterns, such as heat shock proteins, histones, high-mobility group box 1 protein (HMGB1), and S100 molecules, or non-protein damage-associated molecular patterns, including DNA and RNA. Recognition of alarmins is primarily mediated by the NLRP3 inflammasome, which regulates the release of IL-1β and IL-18 (Nürnberger and Kemmerling, 2009; Roh and Sohn, 2018; Relja and Land, 2020).

TLR an essential component of PRRs was initially identified as a gene controlling dorsal-ventral polarity of the Drosophila embryo. However, further research revealed its role in antifungal immunity (El-Zayat et al., 2019). The discovery of these receptors has brought attention to the innate immune system. To this day, 10 human and 13 mouse subtypes of TLRs have been identified. TLRs have the ability to recognize both external PAMPs and internal DAMPs (Duan et al., 2022). These molecules are expressed and present on all cells of the innate immune system including dendritic cells (DCs), macrophage, natural killer (NK) cells, basophils, neutrophils, mast cells, eosinophil, as well as non-immune cells like mesenchymal stem cells (Monlish et al., 2016; Kim et al., 2023). TLR activation leads to the initiation of signaling cascades in the host, which serve as a defense mechanism against invaders and promote the release of inflammatory cytokines and immune modulators to repair damaged tissues (Ali Imani Fooladi et al., 2011). On the other hand, excessive activation of TLR disrupts immune homeostasis leading to the production of pro-inflammatory cytokines and chemokines. This ultimately contributes to the development and progression of various diseases, including sepsis, cancer, and autoimmune diseases (Fitzgerald and Kagan, 2020; Sameer and Nissar, 2021).

Sepsis is one of the most important bacterial infectious diseases and one of the primary factors leading to death on a global scale (Guarino et al., 2023 2023). Despite primary care management, the incidence of severe sepsis rising and mortality rate is noticeably high due to the prevalence of antibiotic resistance. Standard protocol for resuscitation of septic patients is to prescribe antibiotics and prevent organ failure. But in general, no treatment is available to target the underlying mechanism of sepsis (Rhee et al., 2020; Schinkel et al., 2020). The only medication specifically approved for sepsis is recombinant activated human protein C (rhAPC, Xigris^®^, Eli Lilly). However, it was recently withdrawn due to unfavorable findings from the PROWESS-SHOCK trial, which failed to demonstrate a decrease in mortality rate in patients experiencing septic shock at 28 or 90 days (Bernard et al., 2001; Solà et al., 2012; Savva and Roger, 2013). In general, sepsis is caused by an irregular response of the host’s immune system to primary infection, which is characterized by tissue damage and organ failure due to inflammation and suppression of the immune system leading to secondary infection. As previously stated, the recognition of microbial structures (MAMPs) triggers the immune response to infectious agents. This recognition occurs through receptor families that are part of PRR family including nucleotide binding oligomerization domains (NODs)-like receptors (NLRs), c-type lectin receptors (CLRs, including dectin-1, dectin-2, DC-SIGN), RIG-I-like receptors (RLRs, RIG-I, and MDA5), intra-cytosolic DNA sensors, and TLRs (Savva and Roger, 2013; Shekarian et al., 2017). When microbial ligands binds to PRRs, mediators like cytokines are released, which help to control and start the inflammatory reactions required to get rid of pathogens and boost the adaptive immune system (Zheng et al., 2020). TLRs are important in bridging the gap between innate and adaptive immunity. They are capable to identify both invasive pathogens and endogenous threat molecules released from injured or dying cells. TLRs are one of the primary components of the immune response to infectious agents (Medzhitov, 2001). On the other hand, excessive TLR activation may eventually cause an immune system imbalance, raising the possibility of inflammation (El-Zayat et al., 2019). According to studies, various regulatory factors that control TLR activation can also influence the negative feedback of TLR-dependent signaling. Furthermore, TLR signaling pathway antagonists and inhibitors represent a significant therapeutic approach. Accordingly, targeting TLRs is highly significant for the treatment of chronic bacterial and viral infections. Overall, there is a great promise for the prevention and management of infectious diseases with TLR-based targeted therapies (Kawasaki and Kawai, 2014; Anwar et al., 2019; Girkin et al., 2022).

According to the studies, mesenchymal stem cells (MSCs) are TLR-containing non-immune cells. These receptors are one of the effective factors in regulating MSC function (Chen et al., 2013). MSCs are sensitive to altering inflammatory conditions. Accordingly, in sites of damage or inflammation, the exposure of stem cells to TLR ligands causes the activation of TLR-related receptors and the function of these cells is regulated to control the inflammatory response (Pevsner-Fischer et al., 2007; Abdi et al., 2018; Gholizadeh-Ghaleh Aziz et al., 2021). In recent years, stem cell-derived exosomes have received much attention as factors regulating the immune system. The significant advantage of exosomes is that they have many characteristics of their parent cells, and on the other hand, exosomes do not have many of the limitations that exist in the use of cells (Moosazadeh Moghaddam et al., 2023; Essola et al., 2024). Studies have proven that cell receptors such as TLRs can be transferred from cell to cell through exosomes to cause functional changes in the recipient cells. It is also possible to deliver a variety of cellular mediators that regulate the function of cells to the target cells using exosomes as valuable carriers and therefore can be exploited for therapeutic purposes (Li et al., 2022). The HDT approach underpins the therapeutic effects of exosomes on the immune system. The stem cell-derived exosomes function in HDT through immune regulation and modulation, tissue regeneration stimulation, and decreased toxicity to the host (Zhang et al., 2019; Moosazadeh Moghaddam et al., 2023). Accordingly, studies have demonstrated that one of the most significant exosomal cargoes that can control inflammatory responses is microRNA (miRNA). These cargoes target elements of TLR signaling pathways in MSCs or cells that interact with them (Abdi et al., 2018).

Given that TLRs are one of the mediators of interest in the HDT, this review investigates the therapeutic potential of exosomes derived from stem cells against microbial infection based on interactions between exosomes and Toll-like receptors mediated by miRNA cargoes.

## Toll-like receptors

**Figure 2:**
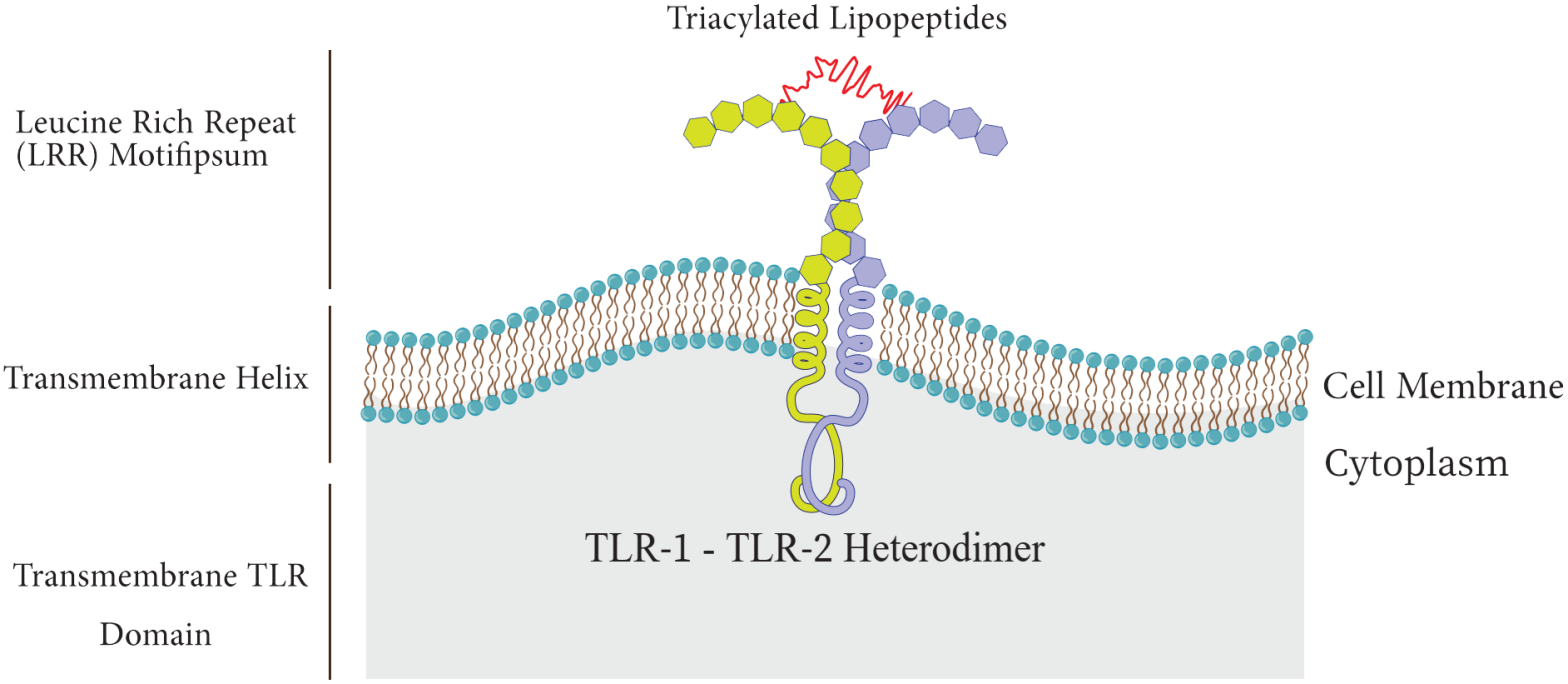
Structure of Toll-like receptor.

Among the PRR family, Toll-like receptors have received more attention and investigation because of their significant function in host defense and involvement in a number of pathological processes, such as acute lung injury (ALI), and sepsis (Lafferty et al., 2010; Vijay, 2018). TLRs are type I transmembrane proteins composed of three domains including I) an extracellular leucine-rich repeat (LRR) domain that mediates recognition of PAMPs, II) a trans-membrane domain, and III) a cytoplasmic Toll-interleukin 1 receptor (TIR) domain which triggers downstream signaling (**Figure 2**) (El-Zayat et al., 2019; Kornilov et al., 2023).

**Figure 3:**
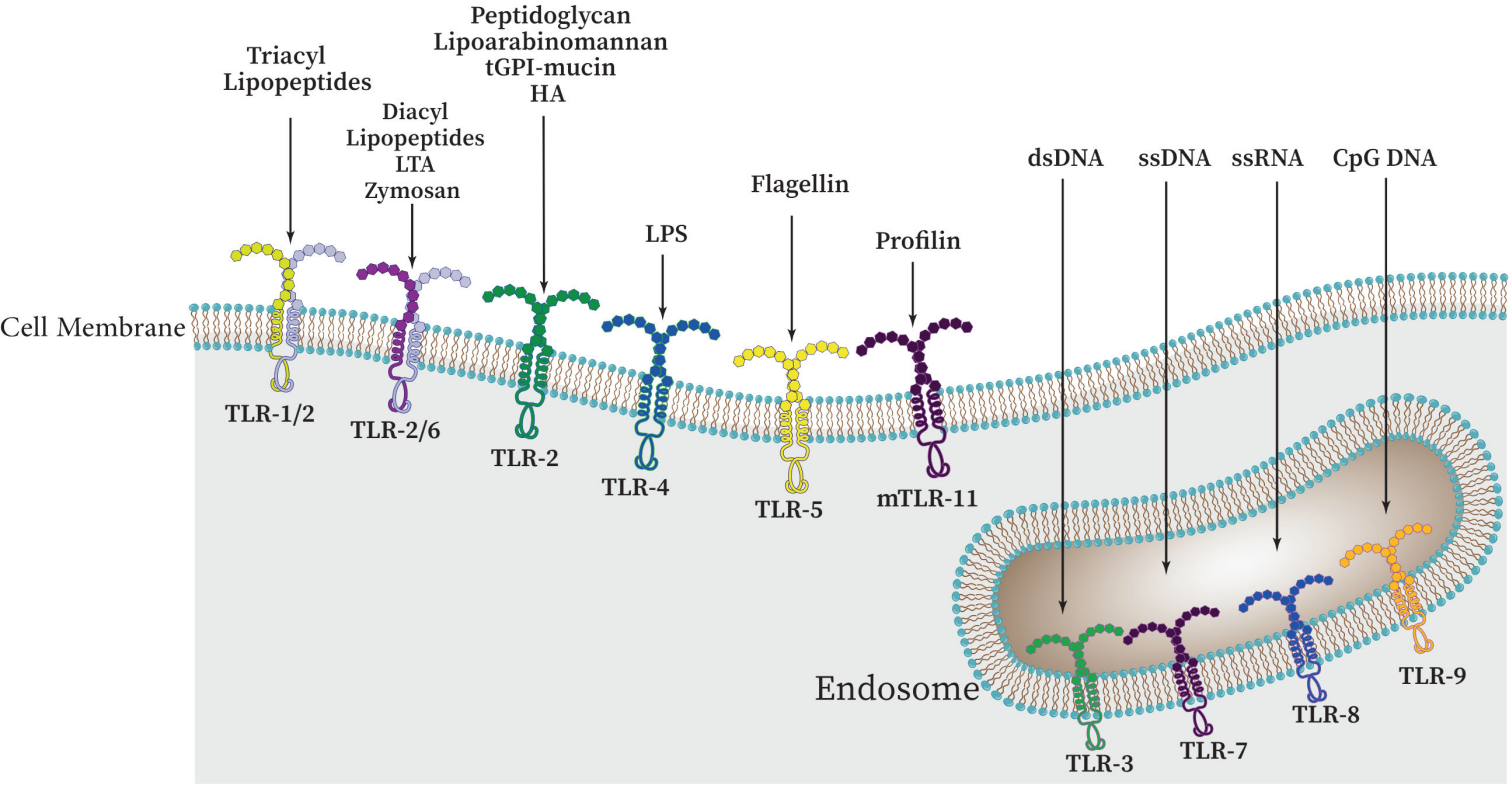
The cellular localization of Toll-like receptors (TLRs) and a variety of PAMPs and DAMPs which can specifically stimulate different TLRs. These receptors can be categorized as follows: TLRs 1, 2, 4, 5, and 6 are present on the outer surface of cells, whereas TLRs 3, 7, 8, and 9 are found within the cell, namely on endosomal membranes. Upon binding to their specific ligands, it is believed that TLRs 3, 4, 5, 7, and 9 initiate signaling through their homodimers. However, depending on the particular ligand involved, TLR2 can form heterodimers with either TLR1 or TLR6.

There are approximately 10 functional TLRs (TLR1-10) in humans and 12 functional TLRs in mice (TLR1-9, TLR11-13; TLR10 is not present in mice). These TLRs are involved in the detection of different microbial products (Kang and Lee, 2011; Xia et al., 2021), the cell surface or intracellular compartments like ER, endosome, endolysosome, lysosome contain TLRs. Therefore, TLRs can be primarily categorized into two distinct subfamilies, namely cell surface TLRs and intracellular TLRs, based on their specific distribution within the cell. While intracellular TLRs are found in the endosome and include TLR3, TLR7, TLR8, TLR9, TLR11, TLR12, and TLR13, cell surface TLRs include TLR1, TLR2, TLR4, TLR5, TLR6, and TLR10 (**Figure 3**) (Chang, 2010; Mielcarska et al., 2021; Giurini et al., 2022).

While intracellular TLRs are primarily capable of identifying nucleic acids derived from bacteria and viruses as well as self-nucleic acids in disease states like autoimmunity, cell surface TLRs are primarily able to identify components of microbial membranes. Research has demonstrated that proper TLR cellular localization plays a key role in controlling signaling, and that downstream of TLRs, cell type-specific signaling determines distinct innate immune responses (Pandey et al., 2015; Duan et al., 2022).

Many microbial agents, including porins, lipoproteins, peptidoglycan, glycoproteins, and glycosylphosphatidylinositol (GPI) anchors from Gram-positive and -negative bacteria, mycobacteria, fungi, mycoplasma, fungi, parasites, and viruses are sensed by TLR2 heterodimers in association with either TLR1 or TLR6 (and possibly TLR10). From Gram-negative bacteria, TLR4 can detect LPS (Farhat et al., 2008; Irvine et al., 2013). After LPS detection, this TLR shuttles to the late endosome to initiate alternative signaling (Oliveira-Nascimento et al., 2012). Flagellin of bacterial flagella is detected by TLR5. In order to detect microbial nucleic acids, TLR3, TLR7, TLR8, and TLR9 are specifically expressed in endosomal compartments. While TLR7, TLR8, and TLR9 recognize unmethylated CpG motif-containing DNA, TLR3 is responsible for identifying viral double-stranded RNA (dsRNA), small interfering RNAs, and self-RNAs generated from damaged cells (Savva and Roger, 2013; Sameer and Nissar, 2021; Kircheis and Planz, 2023). The identification of TLR10 as a sensor for the HIV-1 gp41 protein has been a recent development; however, its precise biological roles in humans remain incompletely understood (Fore et al., 2021; Kircheis and Planz, 2023). TLR11 is localized in the endolysosome and recognizes flagellin, a profilin-like molecule derived from *Toxoplasma gondii*, and an unidentified proteinaceous component of uropathogenic *Escherichia coli* (UPEC) (Qiu et al., 2023). Similar to TLR11, which recognizes profilin from *T. gondii*, TLR12 is primarily expressed in myeloid cells. TLR12 and TLR11 function as a homodimer or a heterodimer (Stejskalova et al., 2023). Also, TLR13 recognizes bacterial 23S rRNA (**Figure 3**, **Table 1**) (Li and Chen, 2012; Goulopoulou et al., 2016). TLR1, TLR2, and TLR4-6 have been primarily been involved in host response to bacterial and fungal infection, so it is not surprising that endosomal TLRs have been primarily assisted in host defenses against viruses (Savva and Roger, 2013; Fitzgerald and Kagan, 2020). Accordingly, endosomal TLRs are mainly involved in the defense against viruses, while TLR1, TLR2, and TLR4-6 are primarily engaged in the host response to infections caused by bacteria and fungi (Savva and Roger, 2013; Goulopoulou et al., 2016). It should be noted that under normal conditions, endosomal TLRs do not respond to endogenous RNAs. It has been demonstrated that self-RNAs carry a number of naturally occurring 2′ modifications including 2′*O*-methyl and pseudouridine modifications, which abrogate TLR-7 and TLR-8 signaling. Additionally, it was also shown that immune stimulation was suppressed by 2′*O*-methyl, 2′-deoxy, and 2′-fluoro substitutions on the ribose backbone of synthetic RNAs suggesting that RNA modifications play a crucial role in the immune escape (Mobergslien and Sioud, 2014; Freund et al., 2019).

**Table 1:** Toll-like receptors, their ligands and origin of ligands (Savva and Roger, 2013).

Toll-like receptor	Cellular localization	Adaptor molecule	Ligand	Source
TLR1 (with TLR2)	Cell Surface	MyD88/TIRAP	Triacyl Lipopeptides Soluble Factors	Bacteria & Mycobacteria *Neisseria meningitidis*
TLR2 (with TLR1 or TLR6)	Cell Surface	MyD88/TIRAP	Lipoproteins & Lipopeptides Lipoteichoic Acid Lipoarabinomannan Peptidoglycan Atypical LPS Phenol-soluble Modulin & Porins Glycoinositolphospholipids & Glycolipids Core and NS3 Proteins, dUTPase & Glycoproteins Beta-glucan & Mannan HSP70	Various pathogens Gram-positive Bacteria Mycobacteria Bacteria *Leptospira interrogans* & *Porphyromonas gingivalis* *Staphylococcus epidermidis* & *Neisseria* *Trypanosoma*, *Toxoplasma* & *Plasmodium* Hepatitis virus, Epstein–Barr Virus & Cytomegalovirus Fungi Host
TLR3	Endosomal	TRIF	Double-stranded RNA	Viruses
TLR4	Cell Surface & Endosomal	MyD88/TIRAP TRIF/TRAM	LPS O-linked Mannan Fusion & Envelope Protein Taxol HSP60 HSP70, Fibronectin & Fibrinogen	Gram-negative Bacteria Fungi Respiratory Syncytial Virus, Mouse Mammary Tumor Virus Plant *Chlamydia pneumoniae* Host
TLR5	Cell Surface	MyD88	Flagellin	Flagellated Bacteria
TLR6 (with TLR2)	Cell Surface	MyD88/TIRAP	Diacyl Lipopeptides, Lipoteichoic Acid & B-glucan	*Mycoplasma*, Gram-positive Bacteria, Fungi
TLR7	Endosomal	MyD88	Single-stranded RNA Imidazoquinoline, Loxoribine & Bropirimine	Viruses & Bacteria Synthetic Compounds
TLR8	Endosomal	MyD88	Single-stranded RNA Imidazoquinoline	Viruses & Bacteria Synthetic Compounds
TLR9	Endosomal	MyD88	CpG-containing DNA Homozoin	Bacteria, Viruses, Fungi *Plasmodium falciparum*
TLR10	Cell surface	MyD88	Lipopeptides (prediction)	–
TLR11	Endosomal	MyD88	Flagellin	Flagellated Bacteria
TLR12	Endosomal	MyD88	Profilin	Apicomplexan Parasites
TLR13	Endosomal	MyD88	23S RNA	Bacteria

The horseshoe-like structure LRR and TLRs interact with their respective PAMPs or DAMPs as a homo- or heterodimer in addition to an accessory or co-receptor molecule to initiate TLR-mediated immune responses (Kawasaki and Kawai, 2014; Bzówka et al., 2023). The activation of signal transduction pathways linked to the activation of NF-kB, IRFs, or MAP kinases is initiated by TLRs upon recognition of PAMPs and DAMPs through the use of TIR domain-containing adaptor proteins such as myeloid differentiation primary response gene (MyD88), TIR domain-containing adaptor inducing interferon (IFN)β (TRIF), and TRIF related adaptor molecule (TRAM) (Deguine and Barton, 2014; Ahmed et al., 2021). The host is ultimately protected from microbial infections by this process, which also controls the expression of chemokines, pro-inflammatory cytokines, antimicrobial molecules and type I IFNs (Deguine and Barton, 2014; Saikh, 2021). TLRs also work with other molecules to identify ligands that are specific to microbes. For instance, TLR2 needs dectin-1, CD36, and CD14 in order to recognize lipopeptides and peptidoglycan, respectively (Behzadi et al., 2021). When it come to recognizing LPS, CD14 is a glycophosphatidylinositol-anchored protein collaborates with TLR4 as a co-receptor (Kumar, 2019). Internalization of TLRs into endosomes is facilitated by ITAM-mediated Syk- and PLCγ2-dependent endocytosis which activates TRIF-dependent signaling. To induce proinflammatory cytokines by TLR7 and TLR9, CD14 is also needed (Baumann et al., 2010; Zanoni et al., 2011). A member of class B scavenger receptor family, CD36 functions as a co-receptor for amyloid-β peptide and oxidized low-density lipoprotein (LDL) (Febbraio and Silverstein, 2007). When a ligand is recognized, Src kinases is activated, resulting in the assembly of TLR4/TLR6 heterodimers. This in turn, causes sterile inflammation which is usually linked to DAMPs. Inflammatory cytokines, ROS and NLRP3 inflammasome activation are also induced (Di Gioia and Zanoni, 2015; Grebe et al., 2018).

Notably, MyD88 also affects other cytokine signaling pathways like IL-1 and IL-18 (Klekotka et al., 2010). TLR3 and TLR4 have the ability to elicit IFN/-reactions by recruiting TRIF. MyD88 is necessary for the synthesis of inflammatory cytokines that promote Th1 responses, whereas, the TRAM/TRIF adapter is generally known to be important for type I interferon production and dendritic cells maturation (Pulendran, 2005; Lundberg et al., 2013).

## TLRs, immunity, and infection

The primary role of TLRs is their broad spectrum pathogen recognition capability. TLRs are present in every immune system cell and these receptors have specific roles in the recognition of PAMP/DAMP by immune responses. The activity of TLRs in innate immunity relies on the production of inflammatory cytokines and the induction of antimicrobial activity (**Table 2**) (Akira and Hemmi, 2003; Piccinini and Midwood, 2010; Hoffman et al., 2023).

**Table 2:** Immune cells and their specific TLR groups, which lead to specific responses of these cells to PAMP/DAMP (Hoffman et al., 2023).

	Toll-like receptors					
	TLR2	TLR4	TLR5	TLR3	TLR7/8	TLR9
**Cell type**	B Cell T Cell Macrophage Dendritic Cell (DC) Monocyte Microglial	B Cell Macrophage Monocyte DC Neutrophil Mast Cell	T Cell Macrophage Monocyte	T Cell Macrophage DC Microglial NK Cell	B Cell Macrophage Monocyte Plasmacytoid DC Myeloid DC	B Cell Macrophage Monocyte Plasmacytoid DC
**Downstream effects**	-Inflammatory cytokines such as TNFα, IL-1β, IL-6, IL-10, IL-12	-Inflammatory cytokines such as TNFα, IL-1β, IL-6, IL-10, IL-12 -TypeI IFN	-Inflammatory cytokines such as IL-6, G-CSF -Chemotactic effects -T Cell stimulation	-Inflammatory cytokines such as IL-6 -TypeI IFN -Chemotactic effects -T Cell stimulation	-Inflammatory cytokines such as IL-12 -TypeI IFN	-Inflammatory cytokines such as IL-1β, IL-6, IL-10, IL-12 -TypeI IFN

When PAMPs or DAMPs activate TLRs, these receptors employ adapter proteins as a platform to recruit TNF receptor-associated factor 6 (TRAF6), GF-beta activated kinase 1/MAP3K7 binding protein 2 (TAB2) and IL-1R-associated protein kinases (IRAK) 1, 2, 4 (Pandey and Agrawal, 2006). As a result of this activity, pro-inflammatory transcription factors, activator protein 1 (AP-1), NF-kB, and interferon regulatory factor 3 (IRF3) translocate (Pandey and Agrawal, 2006; Savva and Roger, 2013). Every transcription factor engages in the transcription of distinct genes that encode various sets of proteins including chemokines (CXCL8 and CXCL10), type 1 interferon (IFN-α, β), antimicrobial peptides, and pro-inflammatory cytokines like tumor necrosis factor alpha (TNF-α), IL-1β, and IL-6 (Gerold et al., 2007).

Human clinical trials and animal models experiments confirm the essential function of TLRs in regulation of infectious diseases (Zhang et al., 2012; Kumar and Barrett, 2022). The primary evidence obtained from the studies that were performed using TLR4 defective C3H/HeJ and C57BL/10ScCr mice. These mice exhibited reduced responsiveness to LPS and were prone to infection of *Salmonella typhimurium* and *Escherichia coli*, which would typically not be lethal (Lee et al., 2005; Hajjar et al., 2012). The importance of TLR signaling in host defense has been highlighted by further studies in mice having knockout TLRs or TLR adapter molecules. Notably, mice lacking TLR2 exhibit high vulnerability to infections caused by *Streptococcus pneumoniae* and *Staphylococcus aureus* (Takeuchi et al., 2000; Echchannaoui et al., 2002). Also, there have been clinical investigations that have linked polymorphisms affecting TLR expression or TLR structure with an increased susceptibility to infection (Mukherjee et al., 2019).

As mentioned, one of the main features of TLRs activation in response to PAMP/DAMP is the control of the secretion of pro-inflammatory cytokines and interferons especially interferons type1 during immune responses (El-Zayat et al., 2019). Cytokines are short-lived and long-range mediators that regulate systemic responses by influencing different tissues (Jaffer et al., 2010). Although TLRs play an important role in host defense against infections through these mediators, TNF-α and IL-1β which are classified as pro-inflammatory cytokines can produce different outcomes in the host, so that in some cases by hyperactivating (cytokine storm), they disrupt the immune system and lead to an exacerbation of systemic damage caused by infection, such as in sepsis and COVID19 (Chousterman et al., 2017; Hu et al., 2021). Cytokine storm, which is a severe elevation in interferons, cytokines and chemokines causes intense inflammation and tissue injury and plays an important role in infectious diseases such as influenza, COVID-19 and sepsis,. The phrase “cytokine storm” was initially employed in 1993 in an published work discussing graft-versus-host disease (GVHD). However, it gained widespread recognition in the field of infectious diseases in 2005, primarily due to its application in H5N1 influenza infection (Clark, 2007).

**Figure 4:**
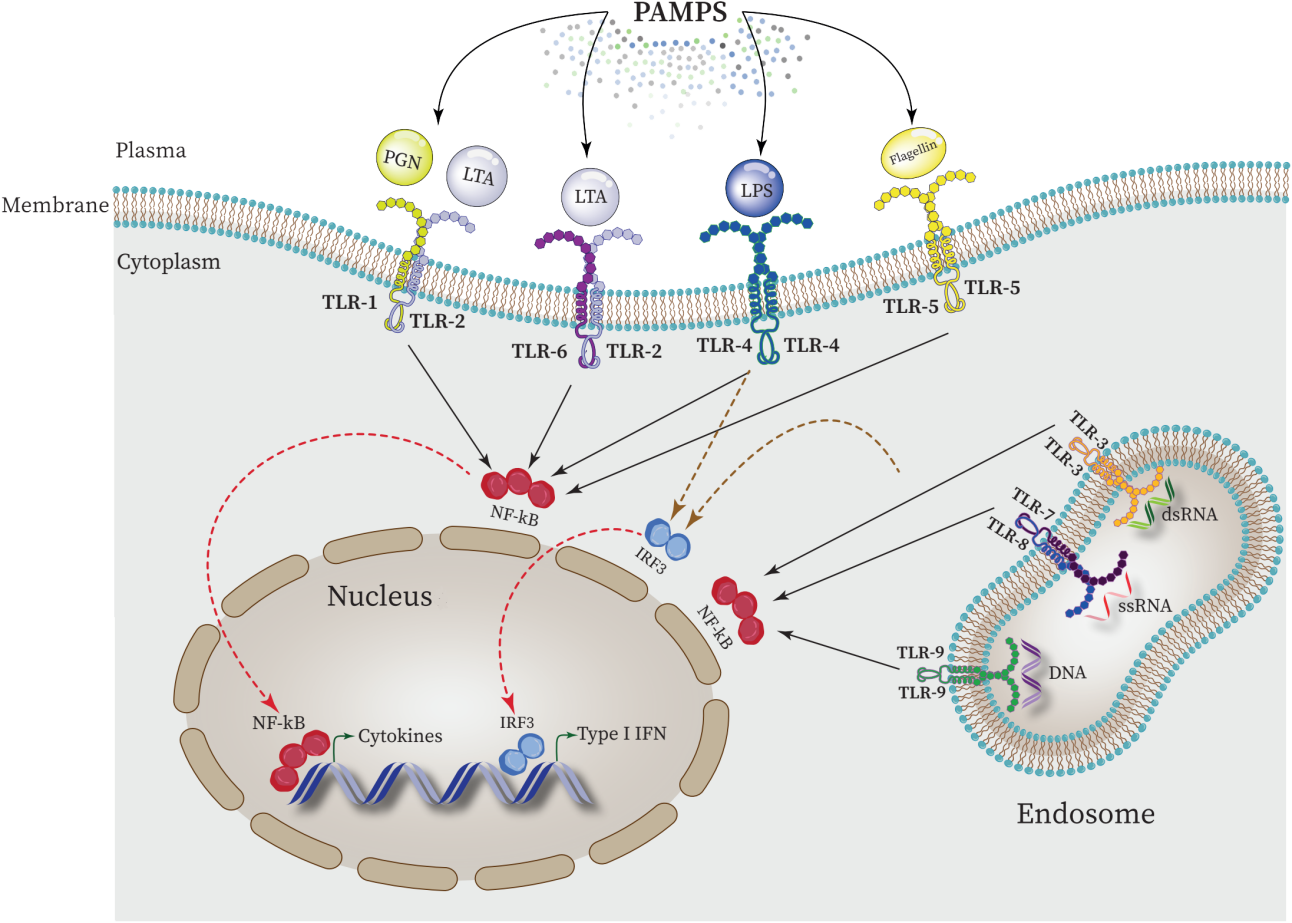
Intraction between PAMPs ligands and TLRs and regulating the expression of proinflammatory cytokines in the nucleus. NF-κB is activated by all TLRs to elevate the expression of proinflammatory cytokines in the nucleus. Furthermore, activation of interferon regulatory factor 3 (IRF3) by some TLRs such as TLR4 leads to upregulation of type 1 INF expression.

Each TLR (TLR4, TLR2, TLR5, TLR9) specifically recognizes bacterial PAMPs including peptidoglycan (PGN), lipoteichoic acid (LTA), lipopolysaccharide (LPS), flagellin, purines, and CpG-DNA. These events activate the nuclear factor κ-light-chain-enhancer of activated B cell (NF-κB) which in turn triggers the expression of genes responsible for the synthesis and release of inflammatory mediators (**Figure 4**) (Zhang and Ghosh, 2001; Dias et al., 2021; Kircheis and Planz, 2023).

In this regard, during cytokine storm, the physical interaction between TLR5 and TLR4 directs the signaling pathway towards MyD88-dependent pathway by forming Myddosome (a complex of MyD88 and IRAK4), which activates NF-κB and the expression of associated downstream pro-inflammatory genes (Kumar, 2020; Tang X. et al., 2023). In general, the activation of TLR signaling in response to DAMPs and PAMPs relies on the signaling pathways mediated by MyD88 and TRIF. This activation subsequently triggers the activation of NF-κB leading to the expression and release of various pro-inflammatory cytokines and other molecules including IL-1α, IL-6, IL-8, TNF-α, reactive oxygen species (ROS), and reactive nitrogen species (RNS) (Yamamoto et al., 2003). Nevertheless, prolonged activation of TLR signaling can give rise to an excessive inflammatory response, potentially causing fetal consequences or triggering autoimmunity which is responsible for the emergence of cytokine storm, systemic inflammation and the progression of disease such as COVID19 and sepsis (Ospelt and Gay, 2010; Manik and Singh, 2022).

On the other hand, a variety of molecules negatively regulate TLR signaling via various mechanisms in order to stop or prevent overactivation of immune system that result in harmful outcomes linked to inflammatory and autoimmune diseases (Wang et al., 2009). Accordingly, suppressor of cytokine signaling (SOCS1; a negative regulator of Janus kinases that functions as a feedback inhibitor of cytokine signaling), peptide-mimetic MyD88 homodimerization inhibitor ST2825, and Casitas B-lineage lymphoma proto-oncogene-b (Cbl-b; an E3 ubiquitin-protein ligase that promotes proteosome-mediated protein degradation) all inhibit the activation of MyD88-dependent pathway (Kawasaki and Kawai, 2014; Wicherska-Pawłowska et al., 2021), and TRAM adaptor with GOLD domain (TAG; a splice variant of the adaptor TRAM, which negatively regulates the adaptor MyD88-independent TLR4 pathway) and selective androgen receptor modulators (SARM) both inhibit the activation of the TRIF-dependent pathway (Ullah et al., 2016). In order to stop these molecules from attaching to downstream molecules or TLRs, they associate with MyD88 or TRIF (Kawasaki and Kawai, 2014).

The role of TRLs in infectious diseases and their function in regulating innate immune responses, in recent years, have drawn the attention of researchers to develop TLR-based drugs to control infections by managing TRLs signaling pathways (Gerold et al., 2007; Duan et al., 2022). As previously mentioned, targeting TLR signaling pathways could be beneficial as a new therapeutic method for infectious diseases control based on host-directed therapy (HDT) (Khan et al., 2016; Kaufmann et al., 2018). In recent years, stem cell-derived exosomes therapy have received much attention as factors regulating the immune system. The therapeutic effects of exosomes on the immune system is based on the HDT strategy (Moosazadeh Moghaddam et al., 2023).

## Exosomes-TLRs intraction and infection control

**Figure 5:**
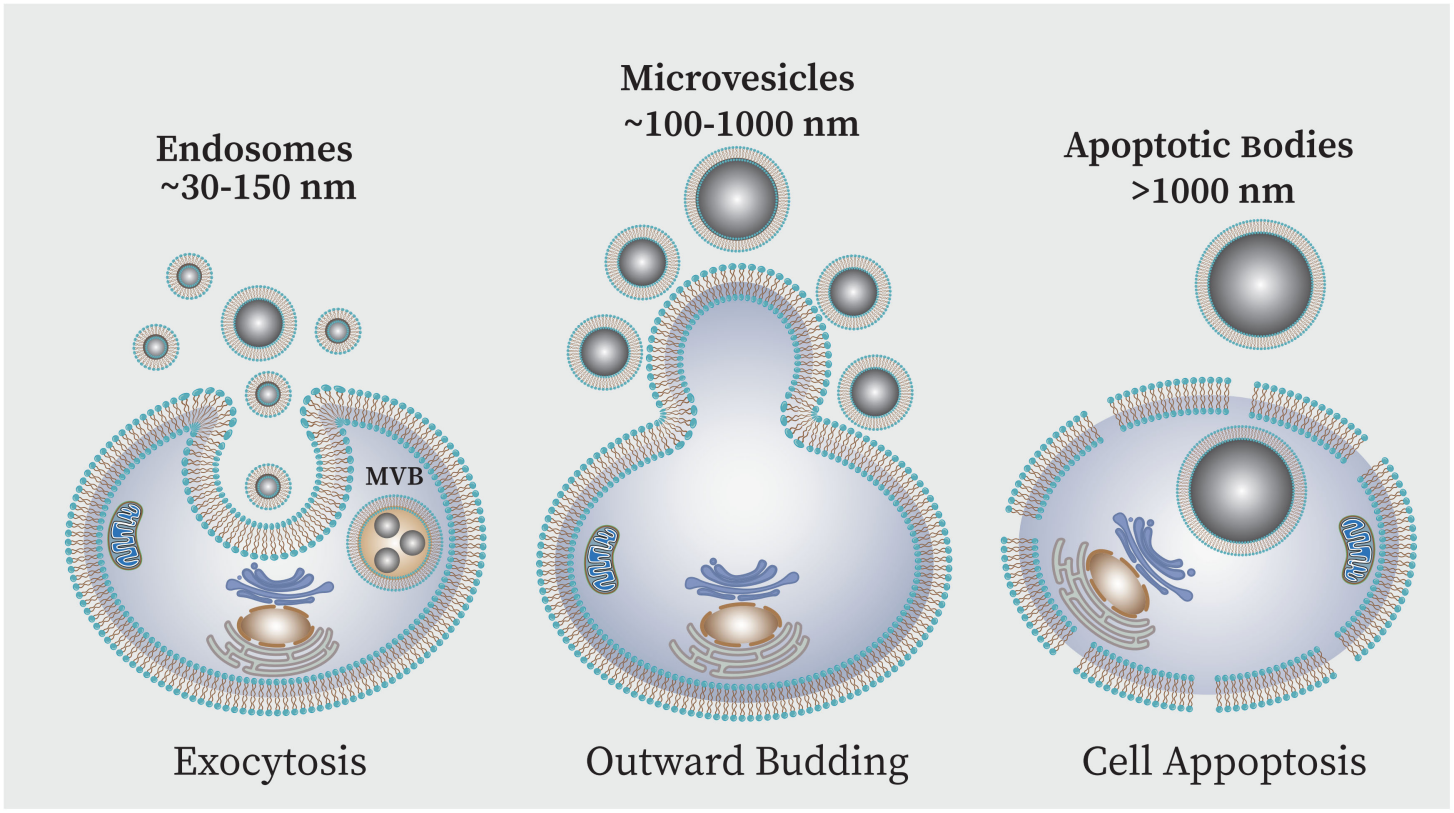
EVs can be classified into three primary subpopulations based on the size and biogenesis process,: (i) exosomes, (ii) microvesicles (MVs), and (iii) apoptotic bodies (AB). Exosomes are produced when endosomes internally budder, resulting in multivesicular bodies (MVBs) which are then released into the extracellular space following fusion with the plasma membrane. Direct outward plasma membrane budding produces MVs. The AB is membrane blebs produced during cell apoptosis.

While cytokines and chemokines are known as mediators of cellular communication to generate and regulate immune responses, recently more complex messengers such as exosomes have been considered (Isola A and Chen, 2017). Exosomes are the smallest extracellular vesicle (EV) structures that can be produced by a wide variety of cells. EVs are generally categorized into three main groups based on size: large-sized apoptotic bodies (1000–5000 nm) are generated during apoptosis; medium-sized micro vesicles (100–1000 nm) secreted from the cell plasma membrane, small-sized vesicles or exosomes (<30–150 nm) produced via the endocytic pathway (Pegtel and Gould, 2019; Moosazadeh Moghaddam et al., 2023). Exosomes vesicles are enclosed in a phospholipid bilayer membrane which originated from the endocytic route and the plasma membrane inward budding results in the formation of these nanovesicles (**Figure 5**) (Kalluri and LeBleu, 2020; Dudzik et al., 2021). These components are essential for intercellular communication because they are present in nearly all body fluids. The components that exosomes carry -i.e. lipids, proteins, metabolites like mRNA, tRNA, microRNA as well as small transcripts of RNA and RNA-protein complexes, chromosomal and mitochondrial DNA-are what give them their significance (Kalluri and LeBleu, 2020). Studies have shown that the elements present in the exosome can be different depending on the type of cells and their physiological conditions. However, some proteins are thought to be particular exosome markers. In this context the tetraspanin family of transmembrane proteins are well-known which includes major histocompatibility complexes (MHC), heat shock proteins (HSP70 and HSP90), and GTPases and CD9, CD81 and CD63 and are among most significant conserved proteins (Kalluri and LeBleu, 2020; Yao et al., 2023).

Exosomes have significant roles in physiology and immunity and have known to play dual roles in both immune system activation and immune suppression (Moosazadeh Moghaddam et al., 2023). However, there are evidences that exosomes generally downregulate immune processes in the absence of stress and pathology (Fleshner and Crane, 2017). Although they are produced by most cells, the exosomes of interest in medicine are stem cell-derived exosomes (stem cell-Exo) due to their immunological features, anti-inflammatory function, regeneration capacity, and antimicrobial function. The benefits of stem cell-Exo also include its low immunogenicity, high stability, small size, lack of tumor genesis, and easier storage needs (Mendt et al., 2019; Nikfarjam et al., 2020). As mentioned, TLRs can be transferred from the stem cells to exosome. Certainly, the presence of these receptors in the exosome is different depending on the type of stem cell from which the exosome originates (**Table 3**) (Shirjang et al., 2017).

**Table 3:** RNA and protein levels of Toll-like receptors in different MSC sources in humans (Shirjang et al., 2017).

Source of MSCs	Gene Expression				Protein Expression
	High	Low	Not significant	Negative	
Bone Marrow	TLR2, TLR3, TLR4, TLR7, TLR8, TLR9	TLR1, TLR5, TLR6, TLR10			TLR1, TLR2, TLR3, TLR4, TLR5, TLR6, TLR9
Adipose	TLR2, TLR3, TLR4, TLR7, TLR9	TLR1, TLR5, TLR6, TLR10		TLR8	TLR1, TLR2, TLR3, TLR4, TLR5, TLR6, TLR9
Umbilical Cord	TLR4, TLR6	TLR1, TLR3, TLR5, TLR9	TLR7, TLR8, TLR10		TLR5, TLR4 with very low expression
Whartons’ Jellay	TLR1, TLR2, TLR3, TLR4, TLR5, TLR6			TLR4	TLR3 under inflammatory conditions

Numerous studies have shown the ability of exosomes derived from MSCs to diminish the advancement and growth of inflammation. This is achieved through the regulation of macrophage polarization by targeting either intracellular signaling pathways or cellular metabolic pathways (Arabpour et al., 2021). MSC-Exos have a specific function where they target the PRRs’ downstream pathway which includes the NF-κB signaling. For instance, in order to induce M2 macrophage polarization, MSC-derived exosomes downregulate the expression of nuclear factor kappa B subunit 1. This was demonstrated by overexpression of M2 markers including arginase-1 (Arg1), IL-10, and decreased levels of M1 marker such as inducible nitric oxide synthase. Based on findings, this activity is related to the microRNAs, especially miR-27a-3p (Wang et al., 2020).

MicroRNAs have obtained considerable interest among exosome biomolecules because of their vital role in regulating gene expression and cellular behavior in diverse physiological and pathological conditions (Zhang et al., 2015). Based on studies, the most prevalent cargo molecules in the exosome are miRNAs, which also play a more useful functional role compared to other cargoes (Hu et al., 2012; Zhang et al., 2015). Numerous studies have demonstrated that the transcriptional responses of TLRs can be regulated by epigenetic processes like DNA methylation, histone modifications and the action of non-coding RNAs like miRNAs, of which miRNAs are of particular importance (Boosani and Agrawal, 2016; Poli et al., 2020). It has been proven that specific miRNA expressions that are upregulated in MSCs give exosomes characteristics like pro-angiogenesis, immunomodulation and anti-apoptosis (Cheng et al., 2020).

In a general description, through targeting of the imperfect antisense complementary regions of both coding and non-coding transcripts, miRNAs are essential elements of posttranscriptional regulation. Recent research has shown that miRNAs regulate gene expression to influence nearly every known cellular processes, including innate and adaptive immune responses (Nejad et al., 2018). The majority of miRNA genes are transcribed by RNA polymerase II, which results in the production of primary transcripts or pri-miRNAs, which are then converted into mature approximately 22-nt miRNA duplexes by RNase III DROSHA and DICER1. Investigations have revealed that the majority of single-stranded miRNAs target the 3’-UTR region of genes; this process is governed by the RNA-induced silencing complex (RISC complex) which controls intracellular pathological processes and biological homeostasis. In other words, miRNAs affect cell behavior and fate that are essential to immune homeostasis by promoting cell activation, polarization, and the development of immunological memory cells in response to environmental stimuli and based on an epigenetic responses (O'Brien et al., 2018).

The majority of miRNAs are either bound to protective proteins including high-density lipoprotein and argonaute protein, or packaged into protective microvesicles like exosomes because naked miRNAs are quickly degraded in the bloodstream (Zhao et al., 2019). Studies have shown that numerous immunomodulatory miRNAs in the human blood are found in exosomes (Fernández-Messina et al., 2015). Transfer of miRNAs to exosomes occurs through an endosomal sorting complexes required for transport (ESCRT)-independent pathway. Thus, it has been demonstrated that the sorting and packaging of exosomal miRNAs in exosomes is related to a signaling pathway that is dependent on neuronal sphingomyelinase 2 (nSMase2) (Nik Mohamed Kamal and Shahidan, 2020; Chen et al., 2021). In addition, it has been shown that RNA-binding proteins are also involved in miRNA sorting, for example Ago2, which controls the secretion of some miRNAs in exosomes by regulating phosphorylation, or heterogeneous nuclear ribonucleoprotein (hnRNP) family proteins, which can induce miRNAs recruitment to exosome by binding to exosomal miRNAs (Groot and Lee, 2020).

As mentioned, studies have demonstrated that miRNAs can influence the signaling pathways associated with the TLRs and lead to the regulation and alteration of the immune responses such as inflammation, cellular infiltration, T-cell activation, and immunity development (He et al., 2014). As a result, miRNA has gained attention recently due to its significance in controlling TLR-dependent inflammation (Momen-Heravi and Bala, 2018). Investigations have shown that GU-rich miRNA sequences, can directly interact with the ribonucleic-acid binding TLR7/8 found in cellular endosomes like exosomes to activate pro-inflammatory signaling pathways in addition to their usual function of controlling gene expression through interaction with cytoplasmic RNA by direct interaction (Nation et al., 2021). So, TLRs-binding miRNA released by cells in damaged or stressed tissues, after being internalized in exosomes and secreted out, in distant recipient cells can generate inflammatory signals (Abdi et al., 2018). Several TLR7/8-binding miRNA including let-7b/c, miR-7a, miR-21, miR-29a/b, miR-34a, miR-122, miR-133a, miR-142, miR-145, miR-146a, miR-208a, and miR-210 have been shown to contribute to inflammation in sepsis, autoimmune, cancer, neurological, and graft-vs-host disease settings thus far (Bosch et al., 2020). Studies demonstrated that these miRNA directly bind to human TLR8 and mouse TLR7. The reports state that TLR7-/- knockout mice are resistant to inflammatory and degenerative effects of TLR-binding miRNA (Fabbri, 2012).

On the other hand, in a study on the role of miRNA in regulation of the immune responses, it has been proven that let-7i miRNA can regulate TLR4 expression in human cholangiocytes by directly binding to TLR4 (Chen et al., 2007). Accordingly, infection caused by *Cryptosporidium parvum* and LPS in cultured cholangiocytes leads to a decrease in let-7 expression through a MyD88/NFkB-dependent mechanism, and this decrease in expression is associated with upregulation of TLR4 (Chen et al., 2007). Subsequently, additional investigations revealed that the 3’ untranslated region of the cytokine-inducible Src homology 2-containing protein is the target of miR-let-7 and miR-98. This results in the translational suppression of this protein in cholangiocytes may be related to the regulation of inflammatory responses in epithelial cells during microbial infection (Chen et al., 2007; Hu et al., 2009). It has also been demonstrated that aberrant miR-155 expression and function are linked to the inflammation and impact immune cell function on several levels by targeting inflammation related genes, including TLRs (Li et al., 2013). The expression of the adapter protein TAB2 in the TLR/IL-1 signaling cascade is repressed by this particular miRNA, thereby playing a crucial role in regulating the feedback mechanism of IL-1β and other inflammatory cytokines produced during LPS-mediated DC activation (Alivernini et al., 2018). Moreover, the suppressor of cytokine signaling 1 (SOCS1) is a target of MiR-155, which can alter SOCS1 transcriptional expression in macrophages activated by LPS (Qayum et al., 2016; Zingale et al., 2021). In addition, miR-155 may regulate TLR3 expression in macrophages via targeting coding sequences of the TLR3 gene. These studies indicate the dual role of miRNAs in enhancing or reducing the level of immune responses due to its interactions with TLRs (Abdi et al., 2018; Zingale et al., 2021).

Cho et al. conducted a study that showed tonsil-derived mesenchymal stem cells (T-MSCs) and T-MSC-derived exosomes can attenuate TLR7-mediated mast cell activation in mice which leads to the reduction of skin inflammation (Cho et al., 2022). The immune system in the skin, which include mast cells, reacts to a variety of environmental and pathological stimuli, such as pathogens (Jiménez et al., 2021). They regulate their function through the expression of TLRs. In their study, the expression levels of inflammatory cytokines were measured after treating the human mast cell line HMC-1 with a TLR7 agonist in the presence or absence of T-MSC exosomes (Cho et al., 2022). The findings demonstrated that in HMC-1 cells treated with TLR7 agonist, the expression of inflammatory cytokines was significantly reduced by T-MSC-derived exosomes containing miRNAs that target inflammatory cytokines. Analysis revealed that in treated HMC-1 cells, the expression levels of IL-8, macrophage colony-stimulating factor (M-CSF), interferon gamma-inducible protein 10 (IP-10), monocyte chemoattractant protein 1 (MCP-1), macrophage inflammatory protein 1 alpha (MIP-1α), MIP-1β, tumor necrosis factor alpha (TNF-α), and tissue inhibitor of metalloproteinases 2 (TIMP-2) were significantly upregulated in the absence of exosomes, however, these expression levels were decreased by T-MSC exosomes (Cho et al., 2022). In addition, the mRNA levels of TLR7 and Myd88, an adaptor that links TLRs to downstream molecules, were evaluated. This was done primarily by comparing the effects of treatment with exosomes derived from T-MSC- or Wharton’s Jelly-MSCs. TLR7 and Myd88 were expressed less frequently in treated HMC-1 cells when exosomes derived from iT- or WJ-MSC were present. However, Myd88 expression was more reduced in T-MSC exosomes than in WJ-MSC exosomes (Cho et al., 2022). In addition, in mice treated with TLR7 agonist, exosomes prevented the growth of both dermal mast cells and CD14-positive cells. The outcomes demonstrated that TLR7 stimulation and mast cell activation under inflammatory conditions are regulated by exosomes derived from T-MSCs (Cho et al., 2022). Consequently, examination of miRNAs expression in T-MSCs revealed that the significant downregulation of inflammatory cytokines in treated HMC-1 cells in the presence of T-MSC exosomes may be associated with the overexpression of miR-214-3p, miR-424-5p, miR-126-5p, and miR-302c-3p (Cho et al., 2022). Apart from these findings, it is noteworthy that WJ-MSCs and their exosomes are the most attractive source of stem cells when immunosuppression is needed in inflammatory or infectious conditions (Drobiova et al., 2023). Research suggests that TLR3 and TLR4 ligation may cause a pro-inflammatory phenotype in bone marrow (BM)- and adipose (AD)-MSCs. In contrast, WJ-MSCs are resistant to TLRs ligation because they can overexpress HGF which is involved in the immune suppression that MSCs mediate, when TLR3 and TLR4 activation is present as well as because they lack expression of several TLRs (Yagi et al., 2010; Sallustio et al., 2019).

The pathological condition known as acute kidney injury (AKI) is characterized by the kidney’s abrupt malfunction. Kidney failure can range from partial loss of kidney function to complete kidney failure (Kellum et al., 2021). Sepsis accounts for 20-50% of cases of AKI in critically ill patients, making it the most common cause in these patients (Poukkanen et al., 2013). Although the pathophysiology of AKI caused by sepsis is complex, however, nephrotoxins, systemic inflammation and hemodynamic changes are the most important influencing factors (Chang et al., 2022). The primary determinant of kidney inflammation severity is thought to be the release of inflammatory cytokines from damaged renal tubular cells. As a result, suppressing the inflammatory response might be essential for treating AKI (Ramesh and Reeves, 2004). Many studies have confirmed the potential therapeutic effects of MSCs on AKI (Sávio-Silva et al., 2020; Liu Y. et al., 2023). For example, human umbilical cord-derived MSCs (hUC-MSCs) have been shown to be highly efficient in repairing cisplatin-induced AKI (Perše and Večerić-Haler, 2022). It has additionally been demonstrated that BM-MSCs have a significant impact on improving kidney function. In this regard, Xiu et al. showed that BM-MSCs could alleviate the inflammation reaction in LPS-induced AKI in rats via downregulation of the level of TLR4 and NF-kB and inhibition of TLR4/NF-kB signaling pathway (Xiu et al., 2018).

On the other hand, in a animal study, Zang et al. demonstrated that exosomes derived from AD- and BM-MSCs can inhibit inflammation and oxidative stress in LPS-induced AKI (Zhang et al., 2022). However, compared to BM-MSCs exosomes, kidney function and structure were significantly improved by AD-MSCs exosomes (Zhang et al., 2022). Based on this, several research has demonstrated that miRNAs and their interactions with TLRs have a significant impact on reducing AKI (Vázquez-Carballo et al., 2021). Liu et al. conducted a study that showed miRNA-342-5p in exosomes secreted from AD-MSCs via TLR9 inhibition leads to the reduction of acute kidney damage (AKI) in LPS-induced sepsis mice (Liu W. et al., 2023). This miRNA facilitates cellular autophagy by downregulating TLR9 expression, thereby leading to protection against sepsis-associated AKI. The results suggest that by influencing necrosis, inflammation, and apoptosis, selective activation of renal proximal tubule TLR9 may worsen ischemic AKI (Liu W. et al., 2023). In addition, Zhang et al. showed that HUC-MSCs-derived exosomes alleviate sepsis-associated AKI via regulating microRNA-146b expression (Zhang R. et al., 2020). They used HUC-MSC exosomes to study the regulatory role of exosomal miR-146b on NF-kB activity and the function of NF-kB on healing the sepsis-associated AKI. Their findings showed that the treatment with hUC-MSC-derived exosomes resulted in downregulation of interleukin (IL)-1 receptor-associated kinase (IRAK1) expression-as the target gene of miR-146b-by increasing the levels of miR-146b. This process leads to the inhibition of NF-kB activity, which ultimately improves the survival of mice with sepsis by controlling and reducing the complications of AKI related to sepsis (Zhang R. et al., 2020).

Another severe inflammatory and traumatic illness that can result in respiratory failure and impaired lung function is acute lung injury (ALI) with a mortality rate exceeding 30% (Huang et al., 2023). ALI can be caused by PAMP ligands such as LPS. According to recent research, autophagy provides protection against ALI (Zhao et al., 2023). Through a lysosome-dependent, regulated process, autophagy is the cell’s natural, conserved method of eliminating unwanted or dysfunctional components. Cellular components are able to degrade and recycle in an orderly manner because of this mechanism (Zhao et al., 2023; Feng et al., 2024). Recently, various studies have shown that administration of hUC-MSCs can lead to reduction of ALI symptoms. Moreover, these cells exhibit a substantial enhancement in survival rates, improvement in anti-inflammatory homeostasis, and diminish in the levels of oxidative stress in LPS-induced acute lung injury (ALI) in vivo (Hong et al., 2020). In a study conducted by Wie et al., exosomes released from h-UC-MSCs were shown to produce autophagy in LPS-induced ALI (Wei et al., 2020). They were able to demonstrate that in LPS-induced ALI, autophagy activation offers protection. The overexpression of miR-377-3p found in hUC-MSCs exosomes is linked to this immunosuppressive effect. TLR4/NF-kB signaling inhibition and downregulation of regulatory-associated with protein of mTOR (RPTOR) significantly reduced lung injury (Wei et al., 2020). According to the studies, RPTOR inhibition prevents hypoxia-induced lung injury by increasing autophagy and decreasing apoptosis (Angara et al., 2016).

In addition, Yi et al. demonstrated that exosomes derived from miR-30b-3p overexpressing-MSCs by serum amyloid A3 (SAA3) inhibition can lead to protection against LPS-induced ALI (Yi et al., 2019). The SAA3 protein is one of the mediators of TLR4 signaling. Based on findings, LPS induced alveolar epithelial cells (AEC) apoptosis could be inhibited by downregulating the expression of SAA3 via overexpressing miR-30b-3p. Overall, SAA3 binding to myeloid differentiation factor 2 (MD-2) activates the NF-kB pathway in a MyD88-dependent manner. When Gram-negative bacteria contain LPS, the TLR4/MD-2 complex recognizes it and triggers an innate immune response (Yi et al., 2019; Jiang et al., 2020).

Also, a study performed by Wang et al. showed that exosomes from AD-MSCs can suppress the progression of chronic endometritis (CE) (Wang et al., 2023). For this purpose, human endometrial stromal cells (hESCs) were used to create an *in vitro* CE and a mouse model with LPS-induced CE in vivo was used to assess the effectiveness of Exos derived from AD-MSCs in vivo (Wang et al., 2023). Generally, CE is an infectious and inflammatory disease associated with long-term, continuous and mild inflammation of the endometrium, which defined by the endometrium’s stromal area becoming infiltrated by plasma cells (Puente et al., 2020). The findings showed that Exos extracted from AD-MSCs can be absorbed by hESCs, whereupon they promote proliferation and prevent apoptosis in hESCs exposed to LPS. Also, these exosomes led to the suppression of TNF-α, IL-6, and IL-1β expression in hESCs. Furthermore, exposure to AD-MSCs-derived exosomes suppressed LPS-stimulated inflammation in vivo. In addition, evaluations showed that endometrial cells, the miR-21/TLR4/NF-kB signaling pathway is how exosome reduce inflammation (Wang et al., 2023). Research has shown that miR-21 expression is considerably downregulated following an LPS challenge, suggesting that miR-21 is involved in the endometrial inflammatory response (Ibrahim et al., 2019). In recent study, it was also shown that exposure to LPS decreased miR-21 levels in hESCs, while administration of AD-MSCs-derived exosomes resulted in increased miR-21 expression (Wang et al., 2023). Based on reports, overexpression of miR-21 inactivates TLR4/NF-kB pathway by downregulation of TLR4 expression (Liu X-J. et al., 2023).

In another study by Wang et al., it was demonstrated that miR-223 in BM-MSCs-derived exosomes contributes to elicit cardio-protection effect in polymicrobial sepsis (Wang et al., 2015). According to the reports miR-223 is highly enriched in MSC-derived exosomes, which may be delivered to cardiomyocytes and cause downregulation of two known targets of miR-223: semaphorin 3A (Sema3A) and STAT3. This prevent sepsis-induced myocardial dysfunction by inhibiting the inflammatory response in macrophages and attenuating cardiomyocyte death during sepsis. Crucially, it has been demonstrated that Sema3A interact with TLRs, particularly TLR4, to start the cytokine storm that is triggered by sepsis (Wang et al., 2015).

In line with these studies, Zhao et al. showed that the poly(I:C), a TLR3 ligand, inhibits miR-143, which enhance immunosuppressive function and the therapeutic effect of MSCs on sepsis (Zhao et al., 2014). Of course, this activity can also be observed in exosomes derived from these cells. In their study, they demonstrated that after treating hUC-MSCs with poly(I:C) as TLR3 ligand, the MSCs’ anti-inflammatory effect on macrophages can be enhanced. Experiments confirmed that expression of miR-143 was greatly decreased in MSCs treated with poly(I:C), and that the expression level of miR-143 could control the impact of poly(I:C) on the immunosuppressive capacity of MSCs (Zhao et al., 2014).

It has been shown that MSC-Exo mediate polarization of macrophages from M1 to M2-like phenotype (Teo et al., 2023). Naive macrophages (M0) can be polarized into two different functional subsets in response to microenvironmental stimuli, pro-inflammatory M1 and anti-inflammatory M2 macrophages (Ruytinx et al., 2018). Pro-inflammatory factors such as TNF-α, IL-1β, IL-6, NO, and proteolytic enzymes, which cause inflammation and increase tissue damage, are produced by M1 phenotype macrophages that are polarized by inflammatory mediators like PAMPs such as LPS. Nonetheless, IL-4 or IL-13 can stimulate M2 macrophages to release immunosuppressive factors such as IL-10, Arg1, transforming growth factor (TGF)-β, IL-1RN, which help to maintain tissue homeostasis and promote healing (Atri et al., 2018; Shapouri-Moghaddam et al., 2018). In addition, observations show that MSC-derived exosomes can lead to immunosuppression by regulatory T cells (Tregs) (Qian et al., 2021). Tregs are able to inhibit proliferation of T cells and cytokine production and they are essential in preventing inflammation related to infectious diseases like sepsis and ALI (Schmidt et al., 2012). Despite the fact that the precise mechanism by which MSCs-Exos affect macrophage polarization remains unclear, it has been demonstrated that the activation of MyD88-dependent TLR signaling pathway, mediated by TLR4, induces the M2 phenotype in both murine and human monocytes through the extra domain A-fibronectin (EDA-FN) (Teo et al., 2023). Research has indicated that miRNAs contribute to macrophage polarization through MSCs-Exos interactions (Essandoh et al., 2016; Self-Fordham et al., 2017). It has been reported that exosomes generated from mouse mesenchymal stem cells use miR-21-5p to induce M2 phenotype of RAW264.7 cells (Liu et al., 2022). Also, exosomes derived from hUC-MSCs enhance M2 macrophage polarization mediated by miR-24-3p (Hou et al., 2023). Similarly, Lin et al. showed that through M2 macrophage polarization, exosomal miR-7704 from mesenchymal stromal cells can modulate a LPS-induced murine ALI model (Lin et al., 2023). According to their findings, the most prevalent and potent miRNA for encouraging macrophages to adopt the M2 phenotype is miR-7704, which also causes M2 polarization by blocking the MyD88/STAT1 signaling pathway (Lin et al., 2023). Intratracheal delivery of exosomes containing miR-7704 proved that these exosomes significantly induced M2 polarization in lung macrophages, thereby effectively restoring lung function and increasing survival (Lin et al., 2023). It should be noted that M1/M2 polarization is determined by the balance of members of STAT (signal transducers and activators of transcription) family. It is commonly acknowledged that one of the primary mediators of inflammatory responses is STAT1. When LPS is bound, TLR4 is usually activated first, which is followed by the phosphorylation of STAT1. Upstream MyD88 activation is necessary for this signal transduction to occur (Tugal et al., 2013; Wang et al., 2014).

Similar to these findings, Ti et al. have also shown that the resolution of chronic inflammation can be achieved through the manipulation of macrophage polarization by LPS-preconditioned mesenchymal stromal cells (LPS pre-MSCs) through the exosome-shuttled miR-let-7b (Ti et al., 2015). In this regard, *in vitro* evaluations for the expression of inflammation cytokines and the distribution of macrophage subtype were performed using human monocytic cell line THP-1, which were treated with LPS pre-hUC-MSCs exosomes and were exposed to high glucose as an inflammatory model. When compared to untreated MSC-derived exosomes (un-Exo), LPS pre-Exo demonstrated a great capacity to modulate upregulation of anti-inflammatory cytokines expression and promotion of M2 macrophage activation (Ti et al., 2015). Exosome analysis revealed the unique presence of miR-let-7b in LPS pre-Exo compared to un-Exo and the potential role of the miR-let-7b/TLR4 pathway in promoting reduced inflammation and macrophage polarization. Additional exploration of the mechanisms governing the expression of miR-let-7b revealed that the regulation of macrophage polarization is significantly influenced by the TLR4/NF-kB/STAT3 signaling pathway (Ti et al., 2015).

In addition, Song et al. demonstrated that exosomal miR-146a enhances the therapeutic efficacy of IL-1β-primed MSCs in the treatment of sepsis (Song et al., 2017). They assessed the therapeutic effects of IL-1β, a pro-inflammatory cytokine that is abundant in the early stages of sepsis, as a pretreatment for hUC-MSCs in a model of sepsis brought on by cecal ligation and puncture (CLP) (Song et al., 2017). Comparing βMSCs to naive MSCs, the findings showed that systemic administration of IL-1β-pretreated MSCs (βMSCs) improved the symptoms of murine sepsis and increased the survival rate more effectively. Moreover, they showed that macrophage polarization towards the anti-inflammatory M2 phenotype can be more effectively induced by the paracrine activity of βMSCs (Song et al., 2017). According to the findings, βMSC-derived exosomes play an effective role in regulating this process. They discovered IL-1β stimulation significantly increased the expression of well-known anti-inflammatory microRNA, miR-146a, which was then specifically packaged into exosomes. Evaluations showed that this exosomal miR-146a upon translocation to macrophages, induced M2 polarization and as a result, improved survival in septic mice (Song et al., 2017). Conversely, when miR-146a is inhibited via transfection with miR-146a inhibitors, the regulatory characteristics of exosomes derived from βMSC are diminished. TLR agonists like IL-1β and TNF-α can trigger the expression of miR-146a, which then suppresses inflammation by targeting immune cells’ IRAK1, TRAF6, and interferon regulatory factor 5 (IRF5) (Song et al., 2017). Moreover, based on reports, high levels of miR-146a has protective effect against ALI by downregulation of TLR4 activity (Luo et al., 2018).

Based on many studies, sepsis is one of the most important complications of bacterial infection, which leads to death in many cases. As mentioned in some of the above studies, the complications of sepsis are influenced by miRNAs and their interactions with TLRs. In **Table 4**, some miRNAs and their intraction and regulatory effect on TLRs in sepsis are listed.

**Table 4:** Some microRNAs related to sepsis and their regulatory effect on infection by affecting the TLR signaling pathway.

miRNAs	Target	Function	Ref
miR-150	TLR2TLR7/TLRs signaling	-Main regulator of immune cell activation and differentiation -Adjusts the TLR2 level by blocking the MY-D88, which ultimately leads to regulation of inflammation.	(Xu et al., 2018; Shomali et al., 2022)
miR-146a	TLR4/TLRs signaling	-miR-146a could reduce the NFkB activation pathway via directly targeting TRAF6, so the production of inflammatory cytokine and chemokines is down-regulated.	(Nahid et al., 2011; Szilágyi et al., 2019)
miR-223	TLRs signaling	-Regulates NF-κB signaling pathway via inhibiting p65 phosphorylation. -The low expression of miR-223 leads to the activation of the NF-κB signaling pathway and production of inflammatory factors.	(Benz et al., 2015; Zhang et al., 2017)
miR-155	TLRs signaling	-The activation of miR-155 leads to the suppression of negative regulators of inflammation. -miR-155 via inhibiting some effectors such SOCS1 and SHIP1 activates NF-KB, which leads to inflammation.	(Shim et al., 2005; Colbert et al., 2017; Pfeiffer et al., 2017)
miR-182	TLR4/TLRs signaling	-The high expression of this microRNA can active TLR recognition via MYD-88 pathway, which causes activation of APC cells. This activation leads to cytokines secretion and activation of Th1 and Th17 cells.	(Meydan et al., 2018)
miR-342	TLR4/TLRs signaling	-miR-342-5p regulates miR-155 positively. -The up-regulation of miR-155 in activated APC cells promote inflammation.	(Vasilescu et al., 2009)
miR-15a	TLR4/TLRs signaling	-miR-15a is involved in TLR4 expression and regulation of macrophage phagocytosis. -The upregulated miR-15a reduces the LPS-induced inflammatory pathways.	(Wang et al., 2018)
miR-574-5p	TLR9/TLRs signaling	-miR-574-5p has a relationship with TLR9. -It directly targets checkpoint suppressor 1 (Ches1) which is involved in TLR9 regulation.	(Li et al., 2012; Zhang Z-J. et al., 2020)
Let-7	TLR4/TLRs signaling	-Let-7a acts as a TLR4 antagonist. -Let-7a is correlated with reduced TNF alpha, interleukin1β, and TLR signaling in an LPS-induced manner. -Levels of let-7a is associated with the regulation of the TLR-mediated immune response in sepsis.	(How et al., 2015; Pfeiffer et al., 2017)
miR-200	TLR4/TLRs signaling	-The miR-200 family are included miR-200b, 200a-429, and miR-200 c-141, which regulate TLR-mediated NF-κB activation. -The miR-200b and miR-200c are involved in regulating TLR4 via downregulation of the MyD88 expression.	(Wendlandt et al., 2012)
miR-149	TLRs signaling	-High expression of miR-149 decreases MyD88 protein expression and production of inflammation inducers such as TNF-α, IL-6, and NF-κB in response to LPS stimulation -miR-149 is able to directly target the 3′-UTR of MyD88 mRNA. -This micoRNA via regulating TLR/MyD88 signaling pathway has an important immunomodulatory role in sepsis pathogenesis.	(Xu et al., 2014)
miR-203	TLRs signaling	-This microRNA negatively regulates TLRs. -The up-regulation of miR203 leads to reduced MyD88 expression, which causes suppression of pro-inflammatory factors -miR-203 changed macrophage polarization from M1 to M2.	(Wei et al., 2013; Arenas-Padilla and Mata-Haro, 2018)

Studies have also shown that exosome/microRNA-based therapies can be a potentially effective treatment against viral infections such as SARS-CoV-2 (Keshavarz et al., 2018; Paul et al., 2022; Mao et al., 2023). During viral infection macrophages as a member of the mononuclear phagocyte system (MPS), play a crucial role in the innate immune response against viruses by bridging the innate and adaptive immune responses via antigen presentation to T cells (Schultz et al., 2021). In COVID-19 infection, activation of macrophages leads to induced production and secretion of type I interferon cytokines such as IL-6 and TNF (Merad and Martin, 2020), which can induce the expression of angiotensin-converting enzyme 2 (ACE2). The ACE2 is a spike protein receptor, which facilities SARS-CoV-2 entry to the macrophage cytoplasm. In this process, the TLRs can recognize viral genetic material such as miRNAs and induce the innate immune response (Loo and Gale, 2011). For instance, SARS-CoV-2 is recognized by TLR7 which is essential in the defense against viral infections by activation of the MyD88 pathways and ultimately increases TNFα, IL-1β, IL-6, IL-12, and IFNα expression. In addition, TLR3 and TLR4 active several factors such as TNF receptor-associated factor, interferon regulatory factor 3 (IFR3), and NF-kB, which finally induce the production of type I, IFNα/β (Iqbal et al., 2020). Furthermore, TLR4 can bind to the spike protein and amplify the inflammatory responses. Studies have shown that host exosomal miRNAs are actively involved in the process of viral infection. In addition, viral miRNAs can also cause extensive changes in the host transcriptome. It was shown that different types of miRNA through TLRs have a vital role in regulating the function of different types of immune cells and host immune homeostasis during viral infection. Therefore, considering the importance of miRNAs in host-pathogen interactions, these small molecules can be considered as modulators of viral infection. In this regards, in a bioinformatics based-study by Schultz et al. (2021), it was investigated whether miRNA cargoes in MSC-derived exosomes have the capacity to modulate the exacerbated cytokines and cell death in severe COVID-19. Accordingly, three datasets of miRNA using different stem cell tissue sources including bone marrow, umbilical cord and adipose tissue were analyzed. Their findings showed that based on cell sources, MSC-derived exosomes can carry different types of miRNA, the most important of which are miR-125a-3p, miR-125b-3p, miR-202-3p, and miR-769-3p. These miRNAs can decrease the production of inflammatory cytokines and minimize cell death by targeting the corresponding 3′-UTRs of multiple mRNAs and TLR pathways such as TLR7/NF-kB signaling. In general, the mechanism of exosomal miRNAs effect on cytokine storm in COVID-19 take place in different ways, the most important of which is to reduce the level of chemokines and pro-inflammatory cytokines through the release of IL-1 receptor agonist, secreting the anti-inflammatory protein TNF-α–stimulated gene 6 protein (TSG-6), and finally, downregulating NF-kB signaling in a TLRs-dependent signaling pathway (Kiaie et al., 2021; Csobonyeiova et al., 2023). On the other hand, it has also been shown that the use of miRNA can be effective in controlling HIV infections. Based on the reports, apart from host miRNAs, the HIV genome encodes viral miRNAs, most of which belong to the trans-activation response element (TAR) group. It has also been shown that exosomal TAR miRNA can upregulate the expression of inflammatory factors, especially IL-6 and TNF-β, which can result from the binding of TLRs to TAR miRNA. This subsequently activates the NF-κB pathway to accelerate the production of cytokines against HIV virus (Sampey et al., 2016).

Finally, although the effective role of stem cells and exosomes in regulating immune responses in infectious diseases has been shown in many studies, a paradigm has been presented by Waterman et al. that shows the polarization effects of TLR3 and TLR4 on the MSCs (Waterman et al., 2010). According to their model, the MSCs developed into two different phenotypes, MSC1 and MSC2, as a result of the activation of these receptors. This theory suggests that the concentrations of two inflammatory mediators, such as TNF-α and IFN-γ, can cause MSCs and subsequently monocytes, to polarize into two different phenotypes (Waterman et al., 2010). When levels of these mediators and LPS as TLR4 specific ligands are lower, MSCs adopt a pro-inflammatory phenotype referred to as MSC1. This leads to the activation of T cell and recruitment of inflammatory cells to the sites of damage by releasing chemokineslike IP-10 (CXCL10), MIP-1α, MIP-1β, RANTES, and CXCL9. TGF-β and IL-6 are always expressed by MSCs. On the other hand, when levels of TNF-α, IFN-γ, and ds-RNA as TLR3 specific ligand, and TGF-β1 are high, MSCs switch to MSC2 phenotype, which promotes the development of Treg. As a result, insufficient levels of TNF-α and IFN-γ appear to enhance the pro-inflammatory phenotypes of MSCs (Waterman et al., 2010). Consequently, the complete molecular cascade of MSCs can take place during various phases of inflammation and still perform their intended role (Gholizadeh-Ghaleh Aziz et al., 2021). MSC2 has a significant function in stimulating the immune system of the host, which thereby completing the cycle of preventing tissue damage and promoting tissue repair. Generally speaking, MSC’s immunomodulatory potential may be increased by TLR3 activation while it may be decreased by TLR4 activation. Therefore, depending on the type of receptors, ligands and cell sources, TLR-priming MSCs can initiate distinct signaling pathways in varying stages of the inflammation and in different types of immune cells. As a result, the hypothesis of Waterman et al. offers a novel perspective on how MSC-based treatments are developed (Waterman et al., 2010). So, further investigations are needed to elucidate how additional pro-inflammatory and anti-inflammatory factors are expressed in the TLR signaling pathway.

## Conclusion

The discovery that exosomes derived from stem cells can exhibit similar functions to stem cells has led to increased interest towards using exosomes in the treatment of diseases based on cell therapy without recruiting stem cells. Due to their small size and lack of replication capacity, exosomes are far less immunogenic and tumorigenic than stem cells. Thus, exosomes are generally more efficacious and safer than stem cells. Exosomes, therefore, have a great deal of potential uses and are frequently employed in stem cell-based therapeutic procedures. Numerous studies have shown that TLRs play a significant role in regulating immune responses during infection based on HDT, and therefore targeting TLR signaling pathways as new approach for controlling infectious diseases has been considered. During infection, it has been demonstrated that stem cells and stem cells-derived exosomes have immunomodulatory and immunosuppressive activities, which in most of them, TLRs are the main mediators. As indicated in this review, one of the most significant exosome cargoes is miRNAs and it has been discovered that these miRNAs frequently regulate TLR activity, which contributes to therapeutic effect of exosomes. Findings have shown that miRNAs in MSCs-derived exosomes have significant effects on inflammatory and non-inflammatory diseases. However, these miRNAs and their downstream pathway/target genes have great diversity. This diversity necessitates comprehensive research on miRNAs and also other cargoes in MSC-Exos. Even though it is commonly known that MSC-Exos can treat infectious diseases, it is crucial to identify which exosome components are most important in order to better understand the mechanism of disease treatment. Furthermore, these exosomes can be altered to carry various cargoes and drugs including miRNAs and their surface-specific receptors can be synthetically modified to facilitate the transfer of exosomes to target cells.

## Author contributions

MM: Conceptualization, Data curation, Investigation, Writing – original draft, Writing – review & editing. EB: Data curation, Investigation, Writing – review & editing. HS: Investigation, Writing – original draft, Data curation. ZG: Data curation, Investigation, Writing – original draft. RK: Investigation, Writing – original draft. MH: Visualization, Writing – original draft. IF: Conceptualization, Supervision, Writing – review & editing.
